# Efficacy and safety of a TIA/stroke electronic support tool (FASTEST) trial: Study protocol

**DOI:** 10.1186/1748-5908-7-107

**Published:** 2012-10-31

**Authors:** Annemarei Ranta, Susan Dovey, Mark Weatherall, Dea O’Dea

**Affiliations:** 1 Department of Neurology, MidCentral Health, Private Bag 11036, Palmerston North, 4442, New Zealand; 2 Dunedin School of Medicine, University of Otago, Dunedin, New Zealand; 3 University of Otago-Wellington, Wellington, New Zealand

**Keywords:** Stroke, Ischaemic Attack, Transient (TIA), Electronic Decision Support (EDS), Decision Support Techniques, Decision Making, Computer-Assisted, Delivery of Healthcare, Integrated

## Abstract

**Background:**

Strokes are a common cause of adult disability and mortality worldwide. Transient ischaemic attacks (TIA) are associated with a high risk of subsequent stroke, and rapid intervention has the potential to reduce stroke burden. This study will assess a novel electronic decision support (EDS) tool to allow general practitioners (GPs) to implement evidence-based care rapidly without full reliance on specialists.

**Methods/design:**

This is a cluster randomized controlled trial comparing TIA/stroke management of GPs with access to the EDS tool versus usual care. The intervention period is 12 months with a 3-month follow-up period for individual patients. Primary outcomes consist of stroke within 90 days of presenting event and adherence to the New Zealand national TIA guideline.

**Discussion:**

A positive study will provide strong evidence for widespread implementation of this tool in practice and has the potential to improve key outcomes for patients and reduce the burden of stroke.

**Trial registration:**

Australia New Zealand Clinical Trials Registry ACTRN12611000792921

## Background

Stroke is the second most common cause of death worldwide and the most common cause of long term adult disability in developed countries [1,2] Stroke costs New Zealand over $450 million every year [3]. If current trends in stroke incidence and mortality continue [4-6], the number of stroke survivors in New Zealand will reach 50,000 by 2015 [7], with overall annual costs of > $700 million. Reducing the burden of stroke is a key goal for health service planning [8].

Transient ischaemic attacks (TIAs) identify people at high risk of stroke. TIA is defined as transient loss of focal cerebral or ocular function lasting <24 hours, attributable to ischaemic vascular disease. TIAs precede stroke in approximately 25% of stroke victims. The 24-hour cutoff point is arbitrary and minor strokes, defined as symptom duration of >24 hours but with subsequent complete or near complete recovery, carry the same high risk of subsequent stroke as TIAs. Both indicate circulatory compromise of brain tissue that is at risk of infarction, but which is yet entirely or nearly entirely salvageable.

Transient cerebral ischemia is typically caused by unstable plaques affecting the larger vessels that supply large amounts of brain tissue. As a result, the majority of strokes that follow TIA or minor stroke are severely disabling or fatal, and these are most likely to occur within 48 hours and up to seven days following TIA while the plaque remains ‘fresh’ [9]. This highlights the importance of urgent intervention to maximize stroke prevention in high-risk patients. A key intervention that reduces subsequent stroke is rapid initiation of best medical therapy via urgent (<24 hour) specialist review [10,11]. Care following this model has been associated with an 80% reduction in 90-day stroke risk from 10.3% to 2.1% (adjusted hazard ratio 0.20, 95% CI 0.08 to 0.49; p = 0.0001) [10].

In New Zealand, providing 24-hour, seven days a week, rapid access specialist TIA clinics is challenging, especially in the smaller district health boards (DHBs), where patient numbers cannot support a sufficient number of neurologists/stroke physicians to staff an around-the-clock specialist service. A UK study has shown that most patients experiencing TIA or minor stroke first seek healthcare from their general practitioner (GP) [12], even when the event occurs outside normal working hours. Thus, in a setting of limited specialist access it seems logical to look for ways that urgent intervention could be offered at the GP level in order to avoid unnecessary and potentially life-threatening treatment delays. However, the scope of clinical conditions managed in general practice is very broad, and individual GPs will have patients presenting to them with TIA/stroke relatively infrequently. Our analysis of general practice records suggests that, on average, a GP will be consulted four to five times per year by a patient presenting with a presumed TIA/minor stroke. However, GP diagnostic accuracy of TIA/minor stroke is only 50% to 80% [13], so the actual rate of recognized TIA/minor stroke patients encountered by GPs may be as few as two to three patients per year.

Electronic clinical decision support systems (EDS) may be especially valuable for assisting clinical decision-making in such circumstances, where a condition is both challenging to diagnose correctly and encountered by generally clinicians relatively infrequently. New Zealand’s MidCentral Stroke Service, in collaboration with the Best Practice Advocacy Centre Inc. (BPAC), has developed a novel TIA/stroke EDS tool. The tool may mitigate the problem of limited or delayed specialist assessment in a setting in which many GPs lack the experience necessary to manage TIA/stroke independently. It aids GPs to accurately diagnose TIAs, promoting treatment initiation at first point of contact rather than awaiting specialist review, and prompts GPs to manage TIA and stroke patients comprehensively and in accordance with New Zealand guidelines [13].

One of the benefits of the EDS tool is that it is inherently educational, providing GPs with immediate feedback on diagnosis and guideline based advice, which can be applied to the management of future patients and improve diagnostic and management skills over time.

Throughout New Zealand, similar BPAC decision support modules are used by 76% of general practices and 85% of GPs. The TIA/stroke module differs from other existing modules because it focuses on the management of an acute medical problem rather than chronic care or disease prevention. However, in its operation it mimics other existing tools. The TIA/stroke tool is a new module that has so far been used only in the MidCentral DHB. National implementation of the TIA/stroke decision support module has the potential to significantly reduce the burden of stroke throughout New Zealand. However, its use may also involve two main possible threats to patient safety: patients may be erroneously diagnosed as not having a TIA and miss out on early treatment and/or specialist referral; and patients may be erroneously diagnosed with a TIA and placed needlessly on potentially harmful medications such as aspirin.

There has been no research investigating impacts of any BPAC EDS modules on clinical behavior and patient outcomes in New Zealand. International evidence on whether point-of-care decision support improves patient care has been equivocal [14,15]. Decision support tools are becoming ubiquitous throughout New Zealand primary care and their utility should be carefully evaluated. In particular, do they assist clinicians to make evidence-based clinical decisions? In a literature review, we identified 175 published reports of research into EDS use in primary care settings, but no tool that addresses the complex clinical care needed for initial presentation of TIA/stroke. Systematic reviews of the decision support literature conclude that there is a shortage of well-designed randomized controlled trials of EDS that provide assessments of its effectiveness in changing patient outcomes, as well as in changing clinician behavior [16-21].

To date, we have conducted four studies to assess the TIA/stroke decision support tool. A pre-launch community-based pilot indicated a high degree of GP satisfaction, excellent guideline adherence when the tool’s management advice was followed closely, and no adverse patient outcomes [22]. A second study comparing stroke experts, GPs, and decision support management in seven sample cases found that management was guideline adherent 33% among GPs without EDS, 92% among specialists, and 100% when the decision support tool was used [23]. Data from a before-and-after study in the MidCentral DHB showed that best medical therapy was in place within 24 hours in 31% of patients before introduction of the EDS system and 52% after its introduction [24]. Finally, a recent unpublished audit of all patients managed using the EDS for the 18 months following its launch has not found any significant adverse events associated with its use. Although reassuring and promising these small non-experimental studies support the need for a well-designed randomized controlled trial to assess this novel treatment approach.

The aim of the FASTEST trial is to formally test the efficacy and safety of New Zealand’s TIA/stroke decision support tool in comparison with usual care. This study has received funding from the Health Research Council of New Zealand (grant 11/268), ethics approval from the Ministry of Health Multi-region Ethics Committee (URA/11/08/048) and has been under way since November 2011.

## Methods/Design

### Design

Cluster randomized controlled trial of general practices with and without TIA/stroke EDS, comparing TIA and stroke management strategies, outcomes, and cost in each arm.

### Intervention

**Figure 1:**
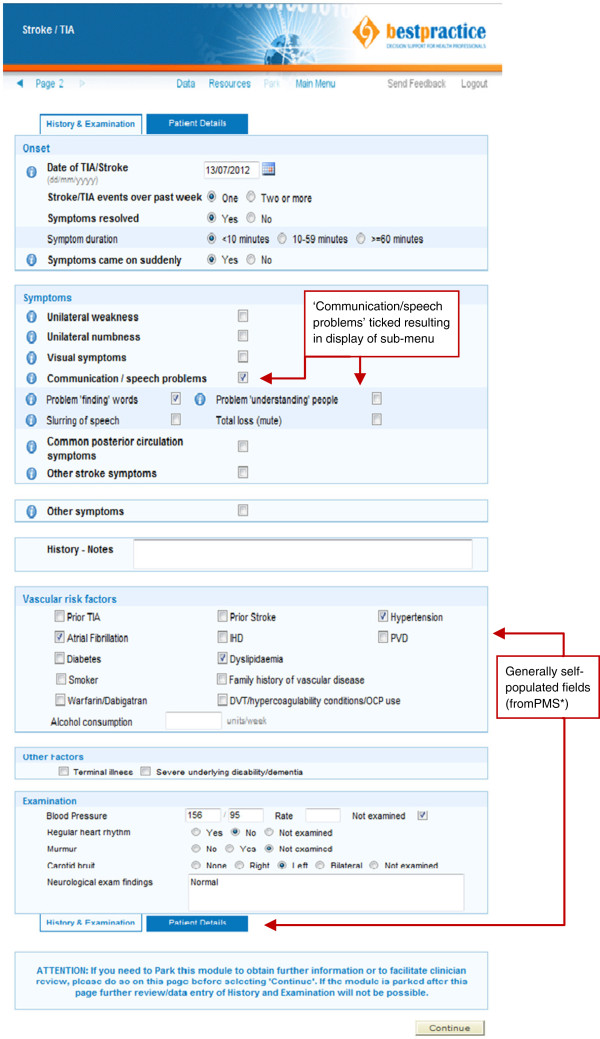
TIA/stroke EDS Data Entry Form with sample case.

The decision support tool is an internet-based module provided and maintained by BPAC. GPs access this tool by clicking a menu button situated on the navigation bar of their practice management software that links them to the BPAC module site. From there they select the TIA/stroke tool from a menu. Once selected, a single page of tick boxes opens up for GPs to complete covering items such as relevant aspects of presenting illness history and a brief focused physical examination. Fields for relevant past medical history (*e.g.*, diabetes and smoking history) are automatically populated by extracting data directly from the practice management system (Figure 1).

**Figure 2:**
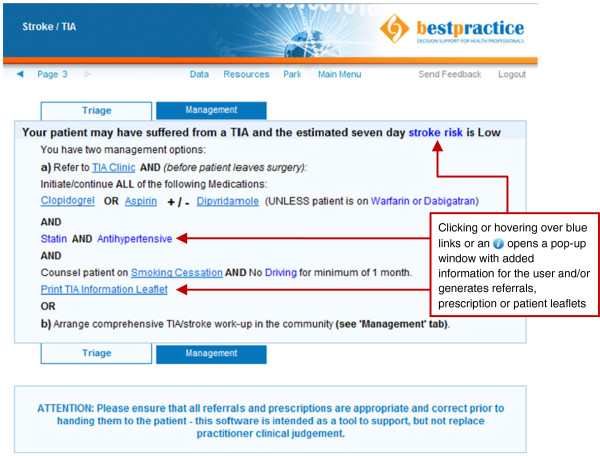
Sample Outcome Page for a low-risk patient with typical TIA symptoms.

**Figure 3:**
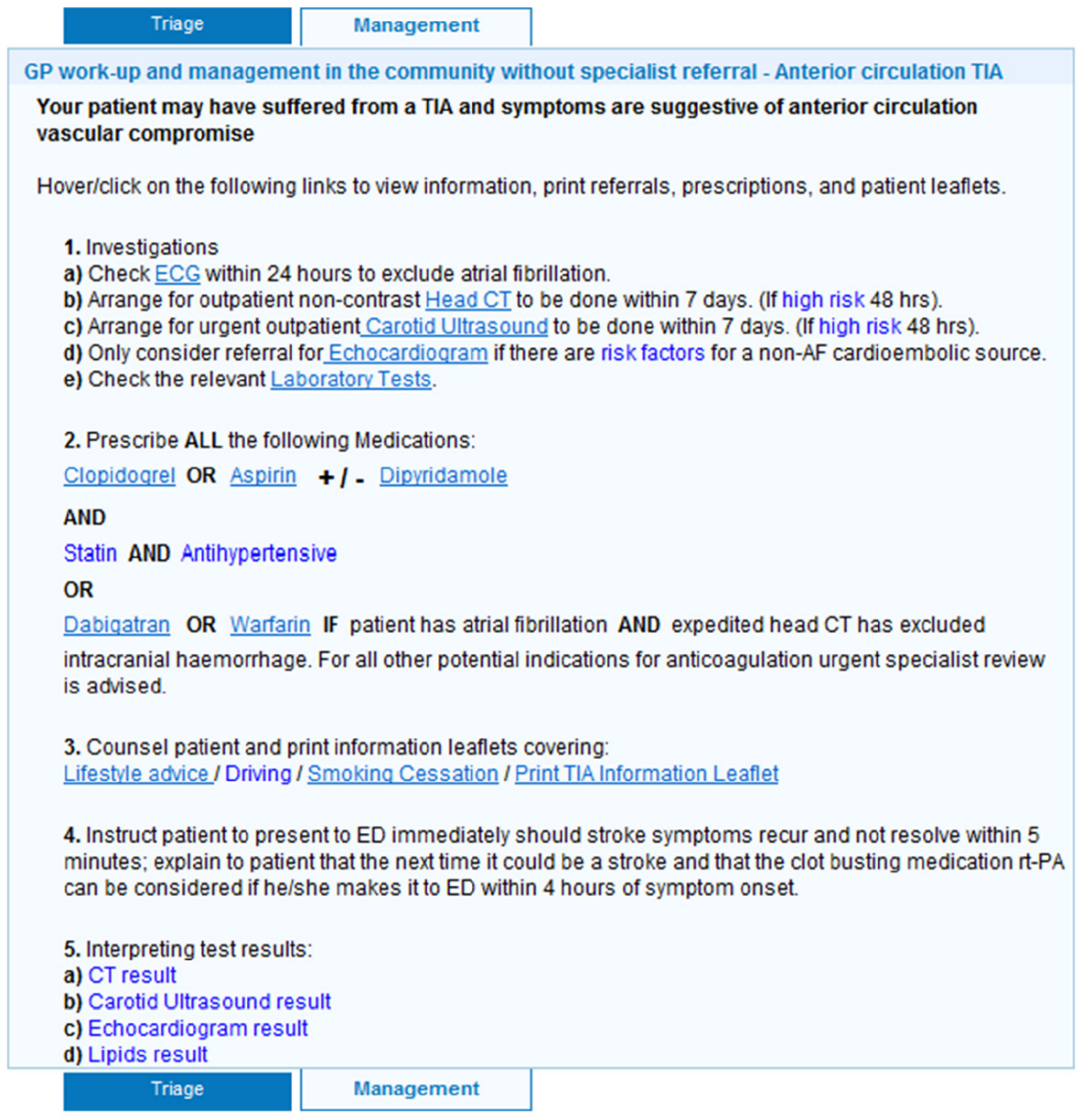
Sample ‘comprehensive TIA/stroke work-up in the community’ instructions for low-risk TIA patient with typical anterior circulation symptoms.

Completing the page of background and clinical presentation data takes approximately two to five minutes depending on the GP’s familiarity with the tool. Based on this information, the software confirms or rejects TIA/stroke as the likely diagnosis. If TIA or stroke is confirmed, a triage recommendation is generated based on the validated ABCD2 risk score [25] supplemented by several other variables taken from the New Zealand TIA guidelines [13] (Figure 2). If patients are triaged into the ‘low risk’ category, GPs are offered the option of either referring them to a TIA clinic for specialist review within seven days or of managing the patients themselves in the community. If community management is selected, a step-by-step outline is provided with links to pre-populated relevant prescriptions, radiology referral forms, and a variety of patient information leaflets (Figure 3). If patients are triaged into the ‘high risk’ category, GPs are advised to refer them to hospital immediately for specialist assessment and diagnostic work-up to be achieved within 24 hours, and GPs are not offered the community management option. However, if a GP feels that urgent hospital referral is not appropriate in any given situation (*e.g.*, the patient refuses to attend the Emergency Department), then GPs have the option to override this recommendation and refer patients to an outpatient specialist TIA clinic instead, as long as a reason for overriding the recommendation is specified. Hospital referral forms are automatically generated and contain all information needed for specialists to prioritize patients appropriately. In the case of a hyper-acute stroke with unresolved symptoms that started within the preceding 4.5 hours (*i.e.*, within the thrombolysis window) the tool is immediately aborted and the GP is advised to call 111 for emergent hospital transfer to a centre where stroke thrombolysis is available.

To preserve patient confidentiality, all patient data for the study generated by the decision support tool are transmitted in an encrypted format to BPAC and from there to the research team.

### Outcome measures

The two primary outcome measure are recurrent stroke within 90 days of initial presentation with TIA or stroke and management in accordance with New Zealand TIA guidelines with regard to: treatment with anti-platelet therapy being achieved within 24 hours of presentation; treatment with statin, anti-hypertensive, and/or warfarin (if applicable and not contraindicated) being achieved as soon as clinically indicated and deemed safe; and receipt of appropriate diagnostic investigations within 24 hours or seven days based on risk stratification.

Secondary outcomes include: adverse events (including, but not limited to, medication side effects, diagnostic delays, and misdiagnoses); occurrence of recurrent TIA, myocardial infarction, major bleeding, and/or death within 90 days; implementation of a comprehensive adjuvant treatment plan (smoking cessation counseling, exercise/diet advice, communication of driving restrictions, and education on thrombolysis); overall treatment cost (including both direct cost relating to treatment of index event and cost of any events related to presenting complaint during the follow-up period); and GP and TIA clinic specialist satisfaction.

### Sample size calculation

The primary outcome is the proportion of participants who have recurrent stroke within 90 days of the presenting TIA/stroke. Based on previous work [9,11,24], we estimate that 10% of patients in the control group will have a stroke or TIA compared to 2% of patients in the intervention group.

Unadjusted for the cluster design, a total sample size of 274, one-half in each of two treatment arms, is needed to detect this size difference with 80% power and type I error rate of 5% [26]. We planned to recruit 40 practices, representing clusters, and used an intra-class correlation coefficient of 0.01 similar to the median intra-class correlation in the paper of Adams [27]. Based on an average of seven participants per practice, the adjusted sample size needed is 292.

This is achievable in 12 months assuming an average of 2.5 GP full-time equivalents per practice and an average of two to three TIA/minor stroke patients per GP per year. The sample size calculation also takes into account an expected 50% to 80% [13] GP diagnostic accuracy. We anticipate that an average of four to five patients will be registered in the study per GP over 12 months, but with an average of only two to three patients having actually suffered a true TIA or minor stroke.

A pre-specified second primary outcome is the proportion of patients who receive management according to New Zealand TIA guidelines. This outcome is likely to be achieved for around 33% of usual care participants compared to an anticipated 92% in the intervention group [23]. Unadjusted for the cluster design, this requires a total sample size of 20 patients. The intra-class correlation coefficient for this outcome will be much higher. Based on a value of 0.4, the study is still likely to have sufficient power to detect this difference.

### Practice engagement

Three districts (Hawke’s Bay and Whanganui in the North Island and the Southern region in the South Island) were chosen to participate in this study. All practices in these areas have access to a hospital based specialist run TIA clinic for consultations during regular hours. All of their practices will have access to 24 hour/7 day-a-week acute medical care in the hospital inpatient setting.

GPs are eligible to participate in the study if their practices use an electronic practice management system and they agree to be randomized into either the intervention group (using the TIA/stroke decision support tool) or the control group (who agrees not to use the TIA/stroke tool) for the 12 months of the intervention phase. All practices in these districts were invited by letter to participate, followed by individual phone calls to promote timely recruitment. GPs were then invited to attend an educational session where the Principle Investigator (PI) reviewed management principles of TIA and stroke and the study’s processes in detail. Representatives from all interested practices attended one of these sessions, confirmed their willingness to participate, signed consent forms, and were randomized on the spot. Posters were provided for practices to have in their waiting rooms, advising patients of the practice’s involvement in the study. Out of the 136 GP practices in these three regions, 44 practices were successfully recruited by the planned intervention start date of 1 March 2012.

A randomization schedule for participating practices was drawn up by the statistical advisor to the project (MW).

After use of the EDS tool, there are some instances when GPs require rapid access to carotid ultrasound or head computed tomography (CT). This is not currently available in the public health system to most GPs enrolled in the trial and related costs for private provision of carotid ultrasound and head CT will be funded through the study to ensure that all patients have similar access to diagnostic tests in the different regions of the study. GP access to these tests endorsed by EDS triage/recommendation is an important aspect of the intervention and has to be assured.

### Study data

GPs in both the intervention and control groups have been provided with a menu button situated on the navigation bar of their practice management software () indicating the link to register patients for the study. GPs will click the button when they encounter a patient they believe to be suffering from a TIA or stroke. In the control group, the button click will prompt patient clinical details to be registered and stored centrally by BPAC and the GP will be advised to continue with routine care. In the intervention group, clicking the button will open up the TIA/stroke EDS module and the GP will then use the software. For each patient in the intervention group, information about diagnosis, triage advice, and management plan given to the GP by the EDS tool will be recorded and stored centrally at BPAC, in addition to patient clinical details.

Throughout the trial and at the end of the 12-month intervention period, records of all patients entered into the BPAC database via the procedure described above will be reviewed for outcome measures three or more months after being registered. GP records will be scrutinized and all data collected will be verified and supplemented by hospital and coroners’ records when applicable. Data collection will be accomplished by a study clinician via use of an electronic Microsoft Access tick box form to facilitate efficient data analysis.

Specific data collected about patients include: final diagnosis including anatomic localization (confirmed by specialist)—TIA, ischaemic stroke, hemorrhagic stroke, or other and anterior versus posterior localization; ABCD2 score if available; initial GP triage destination (community, hospital, or TIA clinic); past medical history of atrial fibrillation, current warfarin use, more than one TIA/stroke event over past seven days (all high risk indicators); medical treatment with antiplatelet(s), statin, and/or antihypertensive accomplished in <24 hours, 24 hours to 7 days, or >7 days; documentation of counseling/education (smoking, diet/exercise, driving, thrombolysis) and by whom it was provided (GP/practice nurse, hospital physician, hospital nurse); investigations and time frame (< 24 hours, 24 hours to 7 days, > 7 days) for ECG, CT head, MRI, echocardiogram, Holter monitor, and carotid ultrasound if obtained; hospital specialist review ‘yes/no’ in < 24 hours, 24 hours, to 7 days, > 7 hours; hospitalizations related to index event ‘yes/no,’ if yes number of days in hospital and discharge location; and TIA, stroke, MI, major bleeding or death within three months of index event, any significant adverse events attributable to medications prescribed after index event, diagnostic delays, and/or misdiagnosis (‘significant event’ is defined as an event that prompted a GP visit/phone call, hospitalization or death). All identifiable data will be expunged from the final dataset to be analyzed for the study, leaving patients identified only by a unique study code.

In addition, at the end of the study, GPs will be surveyed regarding satisfaction with the tool as regards usability, efficiency, and any patient concerns of which they have been made aware. Specialists providing care through a TIA clinic will be surveyed regarding satisfaction with referral quality and any concerns or observations they have made comparing management with versus without TIA/stroke EDS.

### Analysis

This is a single-blinded study with the statistician analyzing the results blinded to the study group of participants. The statistician analyzing the data will be based in a different geographical site from the team managing the study and collecting the data. The analyst will be provided with a data file from which all patient, practice, and study group identifying data has been expunged. These variables will be replaced in the analyst’s file with non-identifiable numeric codes. Before being provided with the data file for analysis, the principal investigator and one other investigator will check the data to ensure that all potentially identifying information has been removed.

The analysis will use a generalized linear mixed model to take account of both the dichotomous outcome variables and the cluster design. These models are more complex than generalized linear models or mixed linear models. The outcomes for individual patients will be the response variables, but we will take account of the cluster design and plan to fit practice and practice-treatment interaction effects as random effects. We may have to explore if the practice-treatment interaction effect has a non-zero value and fit a simpler model. PROC GLIMMIX in SAS will be used to fit the models [28-30].

Analysis of the outcomes will include all patients registered by the GPs, including those who eventually turn out to have a diagnosis other than TIA/stroke (*i.e.*, ‘intention-to-treat’ analysis). However, a pre-specified secondary analysis is of the main outcomes for only patients who were confirmed to have suffered an actual TIA or minor stroke by specialist assessment.

### Cost comparisons

This study is focused on short-term costs and consequences of the proposed intervention, occurring within three months of the initial TIA or stroke.

All costs will be measured as at a specified date. For example, as 'dollars in year 2010 prices,’ or 'year ending in June 2011.’ Costs measured in dollars of another date will be adjusted using appropriate price or cost indices to the chosen 'base date.’ The chosen date will be the latest period, at the time of carrying out the analyses, for which the necessary price or cost indices are available. All costs will be measured excluding Goods and Services Tax (GST). This means that any raw data will be clearly identified as either including or excluding GST, and, in the former case, adjusted to remove GST.

The costs associated with using the intervention (purchase of the computer module from BPAC plus any installation, training, and support costs) will be included in the cost analysis. These costs apply of course to the 'intervention arm' only. Other costs will be collected in tandem with the collection of clinical information, for both 'intervention' and 'control' arms of the trial. That is, costs will be collected for every individual reporting to a practice in the trial with a TIA/stroke. The advantage of collecting data at individual level is that standard deviations and confidence intervals for the cost estimates can then be readily calculated [31]. Also, simulation techniques can subsequently be applied, if desired, to assess the robustness of the conclusions reached from the research.

Costs will be collected for the following: GP visit; ambulance/transport cost; specialist consultation; hospital stay; investigations (ECG, CT, carotid ultrasound, MRI, echocardiogram, Holter monitor, laboratory tests); medications (hospital and ex-hospital).

In general the approach to costing will follow that laid down in Pharmac’s latest (2012 edition) *Prescription for Pharmacoeconomic Analysis*[32]. (Pharmac is the New Zealand agency that decides which pharmaceuticals qualify for government subsidy, and the amount of subsidy). Where applicable 'standard costs' given in the *Cost Resource Manual*[33] provided by Pharmac, such as the average cost per GP consultation, will be used. For consultations and medications any subsidy to patients will be added back to give total cost pre-subsidy. For hospital admissions (day-patient or in-patient), the appropriate diagnosis-related grouping cost-weight will be used and length of stay data will also be collected for collateral information.

## Discussion

The study is challenging, and there are a number of important issues we have attempted to address in the study design.

Study results are intended to be widely applicable nationally and internationally. Involving practices from both North and South Island sites ensures that data will be applicable to a wide range of geographical sites throughout New Zealand. All three study regions are DHBs with relatively small, geographically dispersed populations. On the one hand, this makes them representative of areas that may benefit the most from this type of intervention; yet, our focus on smaller centers may limit application of study data to larger urban centers. To offset this, we have included one DHB centered around a tertiary university medical centre (University of Otago, Dunedin School of Medicine). The three largest population centers in New Zealand (Auckland, Christchurch, and Wellington) were excluded from recruitment because they either have other pathways in place that would take significant time and effort to align with the decision support tool or are in the process of restructuring their services that precluded trial involvement.

Secondly, it can be argued that a cluster randomized design may not be appropriate for this study. However, in the study design we felt that randomization should be at the practice level (cluster design) in order to minimize any potential learning effect created by software use. We decided we should not randomize individual patients because the EDS tool educates GPs on guideline based care and this would affect the care of later patients randomized to the placebo arm and considerably dilute any intervention effect should one exist. Even randomizing GPs to different groups within a practice may have lead to potential confounding because colleagues may discuss cases and their management with one another. GPs within a practice may also cover for one another, which risks inconsistency in EDS use should serial visits occur by a single patient. On the other hand, cluster designs carry the risk of confounding because similar GPs may naturally group themselves in collegial practices. This is addressed in the study design by an increase in sample size to offset the cluster effect.

Another issue is that activation of a ‘registration’ button automatically draws GPs’ attention to the fact that they are participating in a study, which may affect patient management. Furthermore, practices volunteering for the study may be generally more motivated than non-volunteers, which may introduce some degree of selection bias. Both these effects may dilute measured benefit of the intervention because both sources of bias may improve the level of care in the placebo group compared with average GP care encountered in other practices in the country. Thus, while this may make it more difficult for the study to define a significant difference between intervention and placebo, we feel reassured that a false positive trial result is unlikely.

With regard to economic assessments because of the short-term focus of the study, the following items were excluded: lost economic contribution (or lost productivity), *i.e.*, the income from employment lost because of either premature mortality or inability to work because of illness (Pharmac [32] recommends the exclusion of these ‘indirect patient costs’); and the cost of post-hospital institutionalization, nursing care, and social services. These, if included, could be expected, if the intervention is effective, to favor the intervention. In order to get some indication of this effect, discharge location (home, hospital level, or residential home-level care) will be recorded during data collection. Furthermore, lost Quality-adjusted Life-Years (QALYs) will also not be included. Strictly speaking, these are a 'health outcome' rather than an economic cost and will be indirectly captured in the number of strokes occurring in each study arm.

Lastly, the primary outcome of 90-day stroke rate may be difficult to achieve. The study was powered to achieve this outcome, but the only available data on post-TIA stroke rates is now several years old, and the recent more widespread use of secondary preventive medications due to significant efforts toward cardiovascular risk reduction may have had a significant impact on current stroke rates. To account for this potential difficulty, we have selected a second primary outcome in the form of overall guideline adherence.

Overall, we anticipate that information from this study will allow DHBs to decide if wider implementation should go ahead and GPs to decide whether to use the tool. Should the BPAC TIA/stroke EDS tool be found to improve key outcomes for patients then its widespread use has the potential to reduce the burden of stroke in New Zealand.

## Abbreviations

TIA: Transient Ischaemic Attack; EDS: Electronic Decision Support; GP: General Practitioner; DHB: District Health Board; BPAC: Best Practice Advocacy Centre; PI: Principal Investigator; CT: Computed Tomography; ECG: Electrocardiogram; MRI: Magnetic Resonance Imaging; GST: Goods and Services Tax; QALYs: Quality-adjusted Life-Years.

## Competing interests

The authors declare that they have no competing interests.

## Authors’ contribution

AR designed the EDS tool, conceived of the trial, designed the protocol, coordinates the trial and wrote the manuscript. SD contributed to trial design, trial coordination and manuscript preparation. MW contributed to trial design, oversees statistical aspects of the protocol and analysis, and contributed to manuscript preparation. DO designed economic aspects of the trial protocol, assists with analysis and interpretation of economic parameters and edited relevant sections of the manuscript. All authors read and approved the final manuscript.
